# Atherogenic Index of Plasma and Its Association with Risk Factors of Coronary Artery Disease and Nutrient Intake in Korean Adult Men: The 2013–2014 KNHANES

**DOI:** 10.3390/nu14051071

**Published:** 2022-03-03

**Authors:** Hye Ran Shin, SuJin Song, Jin Ah Cho, Sun Yung Ly

**Affiliations:** 1 Department of Food and Nutrition, Chungnam National University, Daejeon 34134, Korea; smile_hrshin@daum.net (H.R.S.); jacho@cnu.ac.kr (J.A.C.); 2 Department of Food and Nutrition, Hannam University, Daejeon 34054, Korea; sjsong@hnu.kr

**Keywords:** atherogenic index of plasma, coronary artery disease, vitamin D, calcium

## Abstract

Coronary artery disease (CAD) has been linked to one of the highest death rates globally. The atherogenic index of plasma (AIP) may be an important predictor of atherosclerosis and cardiovascular disease, superior to the standard atherosclerotic lipid profile. This study investigated the relationship between AIP and obesity indices, blood glucose, lipid profile, and nutrient intake status in Korean adult men. The study included 1292 males aged ≥19 years old who participated in the Korea National Health and Nutrition Examination Survey, 2013–2014. Participants were divided into four groups according to AIP quartiles, calculated as log (triglyceride (TG)/high-density lipoprotein cholesterol (HDL-C)). Body mass index, waist circumference, fasting blood glucose, hemoglobin A1c, total cholesterol, TG, and low-density lipoprotein cholesterol levels increased as AIP levels increased, whereas HDL-C level declined. As the level of AIP increased, intake of saturated fatty acid, calcium, phosphorus, riboflavin, milk, and dairy product decreased significantly, and the contribution rate of milk and dairy products to fat intake decreased. AIP was linked to obesity indices, blood glucose, and blood lipid profile in Korean men, suggesting that it could predict CAD.

## 1. Introduction

Coronary artery disease (CAD) has a high prevalence and incidence, and it is linked to one of the highest death rates in the world [1,2,3]. CAD is an atherosclerotic disease, which is inflammatory in nature, that manifests as stable angina, unstable angina, myocardial infarction, or sudden cardiac death [4]. Major risk factors for CAD include modifiable factors, such as high blood pressure, high blood cholesterol levels, smoking, diabetes, overweight or obesity, lack of physical activity, unhealthy diet, and stress, and the conventional factors, such as age (simply getting older increases risk), sex (men are generally at greater risk of coronary artery disease than females), family history, and race [5].

Serum lipid markers are major risk factors and predictors of CAD and cardiovascular disease (CVD) [2,6]. Dyslipidemia refers to abnormal levels of serum lipids (triglycerides (TG), high-density lipoprotein cholesterol (HDL-C), low-density lipoprotein cholesterol (LDL-C), and total cholesterol (TC)) [7,8]. Therefore, in clinical trials to date, high LDL-C, TG, TC, and low HDL-C have been reported to be highly related to CVD [9,10]. Based on this, there were various indices for predicting CVD, such as the TG/HDL-C ratio [11] and the atherogenic index (AI: LDL-C/HDL-C) [12]. Recently, the Atherogenic Index of Plasma (AIP) was introduced as a better marker for CAD than the conventional ratio in clinical studies [1,2,13,14,15].

The AIP, i.e., the logarithm of the value of TG divided by the value of plasma HDL-C, correlates to LDL-C particle size and esterification rate in apoB-lipoprotein-depleted plasma and is a significant marker composed of TG and HDL-C [16]. AIP may be an important predictor of atherosclerosis and cardiovascular disease, superior to the standard atherosclerotic lipid profile [15]. Many studies have reported that elevated AIP values are positively correlated with diabetes [17,18] and obesity indicators [15]. In addition, high AIP was associated with low blood vitamin D levels in men [19]. Although various studies on AIP are ongoing, domestic studies regarding AIP levels in Koreans are still scarce.

A large-scale study involving approximately 200,000 people in 27 countries for 10 years reported that men had a higher incidence of diagnosed CVD and higher mortality than women [4]. Hence, the male gender is a risk factor for CAD incidence, and mortality in men is thus high. As of 2020, the death rate due to heart diseases in South Korea is the second-highest, following malignant neoplasms among the causes of death, and is the number one cause of death by a single disease. The death rate due to heart diseases per 100,000 persons continuously increases yearly from 55.6% in 2015 to 63.0% in 2020 [20]. In addition, according to the Korea National Health Insurance Corporation, the proportion of the total population of Koreans receiving treatment for CAD was 58.9% men and 41.1% women in 2019. In particular, the medical expenditures for CAD in men increased by 56.2% compared to 2015 [21], indicating an urgent need for preventive measures against CAD in Korean men.

Meanwhile, it is conventionally known that a higher intake of saturated fatty acids is associated with CAD risk [22,23,24]. However, recent studies have reported that milk and dairy products containing saturated fats may prevent CAD [25,26]. Although those studies have not clearly identified how milk and dairy product intake reduces CVD, it is assumed that various factors, such as lactose and calcium, contained in milk and dairy products may prevent CAD. Therefore, it is thought that studies regarding the association between AIP, an indicator of CAD, and dairy product intake in Koreans are required.

This study aimed to investigate the association between AIP and obesity, glucose metabolism, and lipid metabolism indicators in Korean adult men and analyze nutrient and food intakes according to AIP levels using data from the Korea National Health and Nutrition Examination Survey (KNHANES), 2013–2014, the last year in which blood vitamin D levels were measured in Korea.

## 2. Materials and Methods

### 2.1. Participants

**Figure 1:**
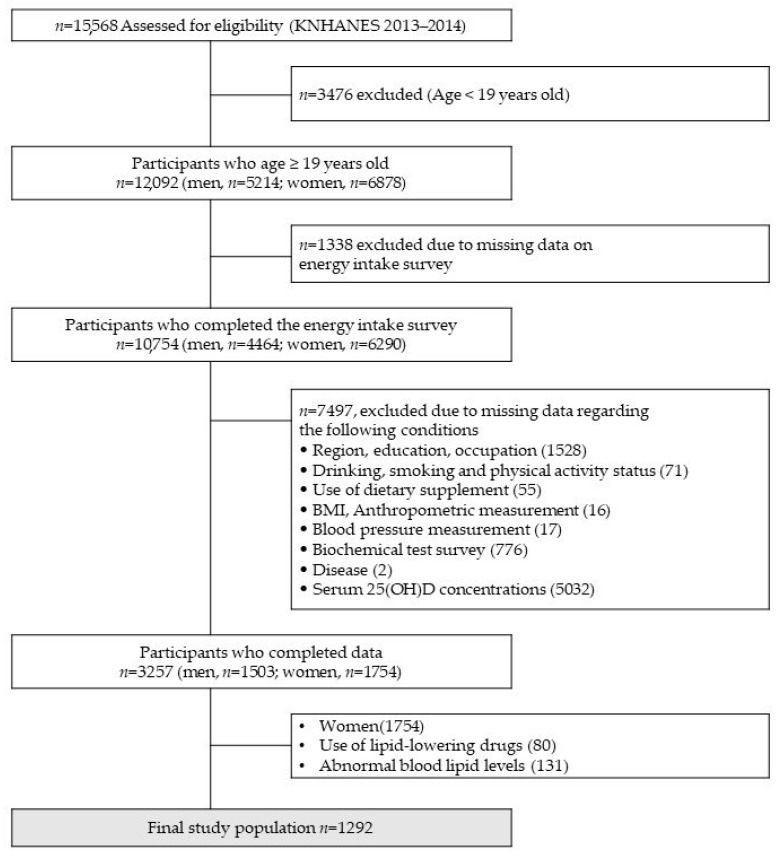
Flowchart of study participant inclusion and exclusion.

The participants in this study were 12,092 persons aged ≥19 years old, from 15,568 who participated in the first and second years of the sixth KNHANES (Figure 1). Among them, 8835 with incomplete information on nutrient and food intake (*n* = 1338) and related data (*n* = 7497) were excluded, and women (*n* = 1754), users of lipid lowering drugs (*n* = 80), and subjects with abnormal blood lipid levels (<1st percentile and >99th percentile), (*n* = 131) were additionally excluded. Finally, data from 1292 adult men were used in the final analysis. The data from the KNHANES used in this study were obtained after receiving approval from the Institutional Review Board of the Korea Centers for Disease Control and Prevention in 2013–2014.

### 2.2. General Characteristics

Age, residential area, occupation, and education level were used as the general characteristic variables of the participants. Age was presented as a continuous variable, and residential area was classified into metropolitan areas (Seoul, Gyeonggi, and Incheon) and non-metropolitan areas. Occupation was classified according to the intensity of physical activity, which included office workers, outdoor workers, and unemployed. Educational level was divided into middle school graduation or below, high school graduation, and college graduation or higher.

Drinking, smoking, physical activity, and dietary supplement intake were evaluated to examine the participants’ lifestyle habits. In terms of drinking status, those who drank alcohol at least once a month for the past year were classified as ‘yes’. For smoking status, those who were currently smoking were classified as ‘yes’. For moderate-to-vigorous physical activity (MVPA), those who participated in at least one high-intensity physical activity (lifting or carrying something heavy, about 20 kg or greater, running, jumping rope, etc., for 20 min or longer per day for 1 week, three times per week), and/or moderate physical activity (performing physical activities, such as carrying light objects, walking briskly, or playing golf, for at least 30 min a day for 1 week, five times per week) were classified as ‘yes’. In terms of dietary supplement intake, those who took a dietary supplement regardless of its type were classified as ‘yes’.

For the disease prevalence rate, KNHANES variables of hypertension, dyslipidemia, stroke, myocardial infarction, angina, diabetes, and renal failure were used and classified into ‘yes’ and ‘no’. In addition to the participants who currently answered ‘yes’, the following participants among the participants who answered ‘no or do not know’ were classified as ‘yes’: hypertension was defined as total systolic blood pressure of ≥140 mmHg or diastolic blood pressure of ≥90 mmHg [27]; dyslipidemia was defined as total cholesterol of ≥240 mg/dL, LDL-cholesterol ≥160 mg/dL, triglyceride ≥200 mg/dL, HDL-cholesterol <40 mg/dL [7]; and diabetes mellitus was defined as ≥6.5% of glycated hemoglobin or fasting blood sugar ≥126 mg/dL [28].

### 2.3. Anthropometric Measurements and Biochemical Indicators

The anthropometric measurements of the participants included height, weight, body mass index (BMI), and waist circumference. Height, weight, and waist circumference were measured in units of 0.1 cm, 0.1 kg, and 0.1 cm, respectively. BMI was calculated as weight (kg) divided by height squared (m^2^). Biochemical indicators, including fasting blood glucose and hemoglobin A1c (HbA1c) as indicators of glucose metabolism; TC, TG, HDL-C, and LDL-C as indicators of lipid metabolism; and blood 25-hydroxyvitamin D(25(OH)D) were analyzed through blood tests after ≥8 h fasting. The TC, TG, HDL-C, and LDL-C values presented as mg/dL values in the KNHANES were converted to mmol/L as follows: mg/dL was converted to the SI (in mmol/L) unit by multiplying the TC, LDL-C, HDL-C values by 0.02586 and TG in mg/dL was converted to the SI unit, by multiplying the TG value by 0.01129 [29]. Men with <1st percentile or >99th percentile blood lipid levels of the same age group were considered abnormal and excluded by referring to the Reference Standard for Korean Health Index published by the National Health Insurance Sharing Service of Korea [30]. The atherogenic index of plasma (AIP) was calculated as a logarithmic transformation of the ratio of TG to HDL-C [16].

### 2.4. Nutrient and Food Intake Survey

In KNHANES, an experienced nutritionist interviewed the study participants and recorded what they ate for 24 h the previous day. Using the 24 h recall data from the KNHANES, this study evaluated the nutrient intake (energy, carbohydrates, fats, proteins, cholesterols, saturated fats, monounsaturated fatty acids, polyunsaturated fatty acids, omega-3 fatty acids, omega-6 fatty acids, dietary fibers, calcium, phosphorus, iron, riboflavin, and the energy ratio of carbohydrate: protein: fat) and food intake by food group (grains, potatoes and starches, legumes and legume products, nuts and seed, vegetables, mushrooms, fruits, meat and poultries, eggs, fishes and shellfishes, seaweed, milk and dairy products, oils and fats, and beverages and alcohols).

Seven major lipid-rich food groups (meat and poultries, grains, oils and fat, milk and dairy products, legumes and legume products, eggs, and fishes and shellfish) and their contribution to fat intake were studied.

Milk and dairy products were classified into milk, ice cream, yogurt, cheese, and cream, and the intake amount of lipids ingested from the food is shown in Table S1. Condensed milk and milk were defined as milk, ice cream, ice milk, and sherbet as ice cream, and yogurt and liquid yogurt were defined and analyzed using the tertiary code of the 24 h recall method among the KNHANES survey data. Raw data were used for cheese and cream.

The nutrient intakes were calculated using the Standard Food Composition Table developed by the Rural Development Administration of the National Institute of Agricultural Sciences (eighth revision) [31]. The statistical analysis of nutrients intake and food group intake were presented as adjusted values for energy intake, age, BMI, smoking, and physical activity. The dietary reference intakes for Koreans in 2020 were used for dietary guidelines for Korean men [32].

### 2.5. Statistical Analysis

All statistical analyses were performed using SPSS version 26.0. The complex sample design of the KNHANES was reflected in the statistical analysis using weight, stratification (kstrata), and clustering (psu) variables. The continuous and categorical variables were presented as mean ± standard error and n (%), respectively. All participants were divided into quartile groups based on their AIP values (<−0.38, −0.38–0.09, 0.09–0.54, ≥0.54). Testing for the significance of trend between the quartiles was presented as *p*-values for trend, using a complex sample general linear model (CSGLM). The general characteristics, except for age and lifestyle habits, were analyzed using cross-analysis of the complex samples. Using the complex samples descriptive statistics, age, anthropometric measures, and biochemical indicators were analyzed. Nutrient intake, with adjustment for energy intake, age, BMI, smoking, and physical activity, was calculated using complex samples, descriptive statistics, and CSGLM. The significance level in all statistical analyses was set to *p* < 0.05.

## 3. Results

### 3.1. General Characteristics of the Subjects According to Quartiles of AIP

The mean age of the subjects in this study was 40.8 years, and the mean AIP was <−0.38 in Q1, −0.38~0.09 in Q2, 0.09~0.54 in Q3, ≥0.54 in Q4. The higher the quartile value of AIP, the higher the average age (*p* for trend < 0.001, Table 1). The number of smokers increased with increased AIP quartiles from Q1 to Q4 (*p* < 0.001), while the proportion of people with MVPA declined (*p* < 0.05). However, there was no difference in residential area, occupation, education level, and dietary supplement use according to the quartile of AIP.

In the disease prevalence rate, hypertension, dyslipidemia, stroke, and diabetes increased from Q1 to Q4 (*p* < 0.05). However, myocardial infarction, angina, and renal failure did not differ between the AIP quartiles.

### 3.2. Anthropometric Measurements Data and Biochemical Indicator According to Quartiles of AIP

As shown in Table 2, height decreased significantly as the AIP quartile increased (*p* for trend <0.05), while weight, BMI, and waist circumference increased significantly (*p* for trend <0.001). As the AIP quartile increased, fasting blood glucose, HbA1c, TC, TG, and LDL-C levels increased significantly (*p* for trend <0.001), whereas HDL-C level decreased significantly (*p* for trend <0.001). Serum 25(OH) D was not significantly different according to the AIP.

### 3.3. Nutrient and Food Intake in Quartiles of AIP

Nutrient intakes adjusted for energy intake, age, BMI, smoking, and physical activity by AIP quartiles are presented in Table 3. The intake of saturated fatty acid (*p* for trend = 0.21), calcium (*p* for trend = 0.001), phosphorus (*p* for trend = 0.014), and riboflavin (*p* for trend = 0.002) decreased significantly with the AIP quartile. There was no significant difference in the protein, polyunsaturated fatty acid, n-3 fatty acid, or dietary fiber intake. Dietary intakes from 14 food groups according to the AIP quartiles were analyzed after adjustment for confounders. Only milk and dairy products consumption decreased considerably from Q1 to Q4 (*p* < 0.05, Table 4).

As a result of analyzing the contribution to fat intake of these seven major fat groups by AIP quartile, the amount of fat consumed from milk and dairy products decreased from the Q1 to the Q4 (*p* for trend < 0.01, Table 5).

Table S1 show the lipid intake of subfoods in milk and dairy food according to quartile of AIP, which were adjusted for age energy intake, BMI, smoking and physical activity. Only milk and lipid intake from milk were significantly decreased from Q1 to Q4 (*p* for trend < 0.01).

## 4. Discussion

This study analyzed coronary artery disease risk factors according to the AIP quartile, using data on adult men extracted from the KNHANES. The AIP, calculated as log_10_(TG/HDL-C), was initially constructed as a biomarker of plasma atherosclerosis. This value is designed to calculate small-dense LDL (sdLDL) [16]. The sdLDL has a high proportion of LDL and is small-sized, more sensitive to oxidative stress, and easily converted into oxidized LDL in the body, which causes inflammatory responses in the sub-endothelium of blood vessels and generates foam cells, leading to atherosclerosis [33]. However, sdLDL analysis has been difficult in clinical practice because it is expensive and requires a complex process. In comparison, AIP can be simply obtained, and accurately reflects sdLDL. Therefore, it has begun to be used as a predictive index for CAD [1]. It has been suggested that an AIP value <0.11 is associated with a low risk of CVD, and values between 0.11–0.21 and >0.21 are associated with intermediate and increased risks, respectively [6].

This study found that low AIP in Korean adult men was favorable for body weight, waist circumference, BMI, fasting blood glucose, HbA1c, and lipid metabolism parameters. Studies by Zhu et al. [15] and Kim [34], in which AIP levels were divided into quartiles as done in this study, reported that as AIP values increased from the first to fourth quartile, BMI values increased. In particular, the study by Zhu et al. found an association between AIP and obesity; when a BMI ≥ 28 kg/m^2^ was defined as obesity, higher AIP levels were positively and strongly associated with obesity [15]. A study by Shen et al. regarding the correlation between AIP and waist circumference stated that an AIP between 0.12 and 0.21 or >0.21 indicated a possibility of borderline abdominal obesity or abdominal obesity, respectively, and that AIP can estimate abdominal obesity [35].

In addition, in this study, the prevalence of hypertension, dyslipidemia, stroke, and diabetes increased as the AIP quartile increased. It was suggested that AIP, which has already been verified as a predictor of CAD in many countries [13,14,15], could be a clinical indicator of vascular and metabolic diseases in Korea as well.

In this study, fasting blood glucose and HbA1c were positively associated with AIP. Furthermore, the prevalence of diabetes increased as the AIP quartile increased. A previous study involving Koreans reported a positive association between insulin resistance and fasting blood glucose levels, even in individuals with normal fasting blood glucose levels [36]. When insulin resistance occurs, it increases the breakdown of free fatty acids in adipose tissue, thereby increasing the amount of free fatty acids flowing into the liver and increasing the synthesis of fat and very-low-density lipoprotein in the liver while developing resistance to the action of insulin that activates lipoprotein lipase in the adipose tissue, which may result in increased blood triglycerides [37]. In addition, HbA1c increases cholesteryl ester transfer from HDL-C, causing a decrease in HDL-C [38]. Therefore, it can be said that there was a significant association between AIP and blood glucose index due to elevated TG and decreased HDL-C. A study by Suh et al. [39] found that the average LDL particle size was smaller and the proportion of sdLDL was higher in patients with type 2 diabetes mellitus (T2DM) compared to that in patients without T2DM [28]; therefore, it is thought that AIP, an indicator reflecting sdLDL, can predict T2DM.

In this study, from Q1 to Q4, the smoking rate decreased while the MVPA rate increased. Blood TG level could be elevated due to reduced physical activity [40] and excessive smoking [41,42]. This high TG value affected the high AIP. Many previous studies have shown that AIP has a positive association with TG, TC, and LDL-C and a negative association with HDL-C [14,15,19]. Traditionally, such lipid components have been used in the prediction of atherosclerotic CVDs, but AIP has the advantage of being a more relevant biochemical parameter than the conventional atherogenic index (AI) [12]. AIP was verified to be an indicator with clinical significance in long-term prognostic observation of diabetes and hypertension through a 9-year longitudinal study [13].

This study attempted to analyze the differences in nutrient and food intake in Korean adult men according to AIP quartiles. A significant difference was observed in saturated fatty acid, calcium, phosphorus, and riboflavin intake, and a higher AIP level was associated with lower intakes of these nutrients. Calcium is notable as an antihypertensive agent, contributing to the inverse association between calcium and CVD risk [43,44]. Inadequate riboflavin intake was associated with increased hypertension and low HDL-C [45].

However, this study found that saturated fat intake was relatively high in those with low AIP values, and it was, thus, necessary to analyze the food sources. The results of a comparison of nutrient intake by food group revealed that lower AIP values were associated with a greater intake of milk and dairy products. Saturated fat, calcium, phosphorus, and riboflavin are all nutrients found in large amounts in milk and dairy products, and their intake, in this study, was proportional to milk and dairy product intake. In addition, we found that as AIP increased, the contribution of fat intake from milk and dairy products decreased.

A study by Vimaleswaran et al. regarding milk intake and cardiometabolic disease outcomes reported that milk drinkers had a 14% lower risk of developing CAD than non-milk drinkers [25]. Dehghan et al. reported that dairy product consumption was negatively associated with death risk due to CVDs or CVD risk. In that study, those who consumed two or more servings/day of total dairy products had a 22% lower risk of major CVD and a 23% lower cardiovascular mortality rate than those who did not consume any dairy products [26]. A multi-ethnic study regarding atherosclerosis reported that a higher intake of saturated fatty acids from dairy products was associated with a lower risk of overall mortality from atherosclerosis and cardiovascular events. That study also reported that a 5 g/day greater intake of milk saturated fatty acid was associated with a 21% lower risk of CVD, whereas a 5 g increase in meat saturated fat intake was associated with a 26% increased risk of CVD [3].

It can be expected that lactose in milk and dairy products may enhance calcium absorption and, thus, that calcium has beneficial effects on CAD. It was reported that when patients with CAD were given an additional intake of 50 g of lactose for 3 weeks, serum cholesterol levels were reduced [46]. Serum TG in mice fed both high fat and lactose was reported to be significantly lower than mice fed a simple high-fat diet [47]. Other potential mechanisms could be influenced by angiotensin-converting enzymes [48], osteocalcin [49] in milk and dairy products, and pathways of intestinal microbial activity, such as gut microbial fermentation [50]. The results of these previous studies suggest that milk and dairy product consumption may help prevent CAD. Our study supports the findings of these previous studies, but further studies are required to determine the mechanisms underlying the lower AIP values with increased consumption of milk and dairy products.

In this study, people with low AIP levels consumed more saturated fat through milk and dairy products; however, excessive saturated fat intake is still considered a major cause of heart disease. Excessive intake of saturated fatty acids is suggested as a potential risk factor for CAD and CVD by increasing LDL-C [22,23,24]. Therefore, the effect of saturated fatty acid intake on CAD according to food ingredients or food groups should be discussed in many studies.

Several epidemiologic and clinical studies show that the intake of omega-3 fatty acids, especially EPA and DHA, helps prevent cardiovascular disease [51,52,53]. In large-scale clinical studies targeting patients with cardiovascular disease or high-risk groups, EPA and DHA could reduce cardiovascular disease mortality [54]. In this study, the intake of omega-3 fatty acids was 1.85~2.05, which was ≥1.4–1.6 g, which is a sufficient intake for Koreans. In addition, the recommended intake ratio of omega-6:omega-3 was 4–10:1, but in this study, it was about 8:1. Currently, it has been observed that omega-3 in Korean men is adequately maintained.

This study had several limitations. Since the results of this study were obtained through a cross-sectional study, it is difficult to derive a causal relationship between diet and AIP. In addition, because dietary data used in this study were based on a 24 h recall, they may not represent individuals’ usual dietary intake. Although people taking lipid-lowering drugs were excluded from the study subjects, it could not be ruled out that other metabolic diseases may affect the relationship between the intake of nutrients and food and CAD risk. However, the strengths of this study are that, to the best of our knowledge, this is the first study to assess the association between AIP and nutrients in Koreans.

This study found differences in coronary artery disease risk factor and nutrient intake according to AIP in Korean men. Those with a low AIP had favorable obesity, diabetes, and lipid metabolic indexes. Those with a low AIP had higher intakes of milk and dairy products, calcium, phosphorus, and riboflavin. AIP was negatively associated with saturated fat consumption attributed to milk and dairy product consumption, although there was no difference in total fat consumption among the AIP quartiles. Prospective studies are needed in the future to investigate the causal relationship between coronary artery disease risk factors and nutrient intake according to AIP. It is also necessary to expand the scope of studies to include both male and female genders for comparative analysis.

## Figures and Tables

**Table 1:** General characteristics of the subjects according to quartiles of AIP (Atherogenic Index of Plasma).

		Quartiles of AIP				Total (*n* = 1292)	*p*-Value
		Q1 (*n* = 322) <−0.38	Q2 (*n* = 324) −0.38 to 0.09	Q3 (*n* = 323) 0.09 to 0.54	Q4 (*n* = 323) ≥0.54		
Age (years)		36.7 ± 0.80	40.7 ± 0.78	42.5 ± 0.88	43.1 ± 0.81	40.8 ± 0.38	<0.001 ^(1)^
Residential area	Metropolitan	154 (51.5)	156 (51.2)	171 (55.2)	176 (57.9)	657 (53.8)	0.374
	Non-metropolitan	168 (48.5)	168 (48.8)	152 (44.8)	147 (42.1)	635 (46.2)	
Occupation	Office workers	86 (27.1)	90 (29.8)	99 (36.1)	107 (36.0)	382 (32.1)	0.242
	Outdoor workers	161 (49.7)	159 (46.2)	149 (42.4)	152 (19.6)	289 (22.1)	
	Unemployed	75 (23.2)	75 (24.0)	75 (21.5)	64 (22.8)	339 (24.4)	
Education level	<Middle school	62 (14.1)	71 (15.1)	73 (15.9)	72 (17.5)	278 (15.6)	0.767
	High school	132 (46.2)	129 (43.7)	120 (39.9)	124 (41.5)	505 (42.9)	
	≥College	128 (39.7)	124 (41.1)	130 (44.2)	127 (40.9)	509 (41.4)	
Drinking	Yes	249 (80.0)	231 (70.9)	243 (75.8)	248 (76.8)	971 (75.9)	0.096
	No	73 (20.0)	93 (29.1)	80 (24.2)	75 (23.2)	321 (24.1)	
Smoking	Yes	101 (31.0)	116 (36.6)	150 (48.4)	163 (53.7)	530 (40.9)	<0.001
	No	221 (69.0)	208 (60.4)	173 (51.6)	160 (46.3)	762 (57.1)	
MVPA ^(2)^	Yes	105 (33.4)	84 (28.4)	68 (23.2)	67 (22.2)	324 (27.0)	0.019
	No	217 (66.6)	240 (71.6)	255 (76.8)	256 (77.8)	9686 (73.0)	
Dietary supplement	Yes	131 (38.6)	130 (37.3)	117 (35.1)	134 (40.9)	512 (38.0)	0.586
	No	191 (61.4)	194 (62.7)	206 (64.9)	189 (59.1)	780 (62.0)	
Hypertension	Yes	51 (10.1)	78 (18.5)	92 (26.4)	105 (29.7)	326 (20.9)	<0.001
	No	273 (89.9)	246 (81.5)	231 (73.6)	218 (70.3)	966 (79.1)	
Dyslipidemia	Yes	11 (3.2)	49 (15.0)	100 (30.5)	271 (83.0)	431 (32.4)	<0.001
	No	311 (96.8)	275 (85.0)	223 (69.5)	52 (17.0)	861 (67.6)	
Stroke	Yes	1 (0.3)	2 (0.3)	4 (0.5)	7 (1.8)	14 (0.7)	0.034
	No	321 (99.7)	322 (99.7)	319 (99.5)	316 (98.2)	1278 (99.3)	
Myocardial infarction	Yes	-	1 (0.2)	3 (0.8)	1 (0.3)	5 (0.3)	0.267
	No	322 (100)	323 (99.8)	320 (99.2)	322 (99.7)	1287 (99.7)	
Angina	Yes	4 (0.8)	1 (0.1)	3 (0.4)	4 (1.1)	12 (0.6)	0.178
	No	318 (99.2)	323 (99.9)	320 (99.6)	319 (98.9)	1280 (99.4)	
Diabetes mellitus	Yes	15 (2.6)	27 (6.5)	30 (7.4)	53 (13.5)	125 (7.4)	<0.001
	No	307 (97.4)	297 (93.5)	293 (92.6)	270 (86.5)	1167 (92.6)	
Renal failure	Yes	-	-	1 (0.3)	2 (0.4)	3 (0.2)	0.454
	No	322 (100)	324 (100)	322 (99.7)	321 (99.6)	1289 (99.8)	

^(1)^ *p* for trend. ^(2)^ MVPA (moderate-to-vigorous physical activity): ‘yes’ for physical activity—moderate (for more than 30 min at a time and more than five times per week) and/or physical activity—high (for more than 20 min at a time and more than three times per week).

**Table 2:** Anthropometric measurements data and biochemical indicators according to AIP quartiles.

	Quartiles of AIP				Total (*n* = 1292)	*p* for Trend
	Q1 (*n* = 322) <−0.38	Q2 (*n* = 324) −0.38 to 0.09	Q3 (*n* = 323) 0.09 to 0.54	Q4 (*n* = 323) ≥0.54		
Body mass index (kg/m^2^)	22.5 ± 0.18	23.7 ± 0.20	25.0 ± 0.20	25.7 ± 0.21	24.27 ± 0.10	<0.001
Waist circumference (cm)	78.0 ± 0.50	82.2 ± 0.52	85.1 ± 0.52	87.8 ± 0.53	83.48 ± 0.26	<0.001
Fasting blood glucose (mg/dL)	92.1 ± 0.66	95.6 ± 1.12	97.8 ± 0.90	104.3 ± 1.39	97.51 ± 0.51	<0.001
HbA1c (%)	5.54 ± 0.03	5.67 ± 0.04	5.72 ± 0.03	5.87 ± 0.05	5.70 ± 0.02	<0.001
TC (mmol/L)	4.53 ± 0.04	4.79 ± 0.05	4.85 ± 0.04	5.08 ± 0.05	4.81 ± 0.02	<0.001
TG (mmol/L)	0.70 ± 0.01	1.15 ± 0.01	1.62 ± 0.02	2.82 ± 0.05	1.57 ± 0.01	<0.001
HDL-C (mmol/L)	1.51 ± 0.01	1.31 ± 0.01	1.16 ± 0.01	1.07 ± 0.01	1.26 ± 0.01	<0.001
LDL-C (mmol/L)	2.87 ± 0.04	3.26 ± 0.04	3.36 ± 0.04	3.44 ± 0.04	3.23 ± 0.02	<0.001
Serum 25(OH)D (ng/mL)	17.4 ± 0.42	17.3 ± 0.48	17.0 ± 0.44	16.8 ± 0.40	17.17 ± 0.25	0.216
AIP	−0.79 ± 0.02	−0.13 ± 0.01	0.32 ± 0.01	0.93 ± 0.02	0.08 ± 0.01	<0.001

TC: Total Cholesterol; TG: Triglycerides; HDL-C: High-density lipoprotein cholesterol; LDL-C: Low-density lipoprotein cholesterol.

**Table 3:** Daily nutrient intake in quartiles of AIP (adjusted energy intake, age, BMI, smoking, and physical activity).

		Quartiles of AIP				Total (*n* = 1292)	*p*-Value	*p* for Trend
	Reference Value for KDIRs ^(1)^	Q1 (*n* = 322) <−0.38	Q2 (*n* = 324) −0.38 to 0.09	Q3 (*n* = 323) 0.09 to 0.54	Q4 (*n* = 323) ≥0.54			
Energy (kcal)	1900–2600	2561.8 ± 66.7	2623.0 ± 79.3	2561.2 ± 72.8	2620.6 ± 70.6	2591.7 ± 41.2	0.834	0.706
Carbohydrate (g)	130	361.9 ± 5.87	362.2 ± 6.18	361.4 ± 5.93	346.1 ± 6.75	357.9 ± 3.20	0.221	0.089
Lipid (g)	-	60.7 ± 1.72	58.4 ± 1.99	59.1 ± 1.81	57.1 ± 1.59	59.8 ± 1.04	0.469	0.456
Protein (g)	60–65	88.2 ± 1.55	87.4 ± 2.22	87.7 ± 1.72	90.1 ± 1.94	88.4 ± 1.02	0.764	0.154
Cholesterol (mg)	<300	331.1 ± 15.1	327.9 ± 16.7	319.9 ± 14.7	321.7 ± 18.2	325.1 ± 9.43	0.948	0.643
Saturated fatty acid (g)	<7% of energy intake	17.9 ± 0.65	17.2 ± 0.73	17.2 ± 0.58	15.8 ± 0.57	17.0 ± 0.36	0.076	0.021
Monounsaturated fatty acid (g)	-	19.3 ± 0.65	19.0 ± 0.82	18.7 ± 0.69	17.7 ± 0.61	18.7 ± 0.40	0.300	0.075
Polyunsaturated fatty acid (g)	-	14.8 ± 0.56	13.6 ± 0.58	14.6 ± 0.61	15.2 ± 0.65	14.6 ± 0.33	0.233	0.411
Omega-3 fatty acid (g)	1.4–1.6 ^(2)^	1.96 ± 0.09	1.73 ± 0.89	2.00 ± 0.13	2.14 ± 0.14	1.96 ± 0.06	0.058	0.127
Omega-6 fatty acid (g)	9–13 ^(2)^	13.0 ± 0.51	12.0 ± 0.52	12.7 ± 0.53	13.2 ± 0.56	12.7 ± 0.29	0.354	0.558
Dietary fiber (g)	25–30	26.0 ± 0.74	25.9 ± 0.70	25.5 ± 0.62	25.1 ± 0.62	25.6 ± 0.37	0.800	0.329
Calcium (mg)	700–800	614.6 ± 21.6	569.3 ± 18.7	552.7 ± 16.3	520.8 ± 17.6	564.3 ± 10.5	0.012	0.001
Phosphorus (mg)	700	1348.1 ± 22.7	1311.9 ± 19.7	1287.5 ± 21.2	1281.4 ± 19.5	1307.2 ± 11.9	0.089	0.014
Iron (mg)	9–10	19.1 ± 0.67	21.0 ± 0.97	21.1 ± 1.28	23.2 ± 4.65	21.1 ± 1.50	0.444	0.433
Riboflavin (mg)	1.3–1.5	1.80 ± 0.49	1.66 ± 0.40	1.64 ± 0.41	1.60 ± 0.38	1.67 ± 0.23	0.013	0.002
	C:P:F ratio (%)							
CHO (%)	55–65	62.8 ± 0.67	63.5 ± 0.76	0.63 ± 0.69	62.2 ± 0.74	62.9 ± 0.38	0.592	0.510
Protein (%)	7–20	15.0 ± 0.27	15.0 ± 0.36	14.8 ± 0.28	15.7 ± 0.31	15.1 ± 0.16	0.145	0.111
Fat (%)	5–30	22.1 ± 0.56	21.4 ± 0.58	21.8 ± 0.53	21.9 ± 0.59	21.8 ± 0.32	0.804	0.953

^(1)^ Recommended intake. ^(2)^ Adequate intake.

**Table 4:** Food intake in quartiles of AIP (adjusted for energy intake, age, BMI, smoking, and physical activity).

	Quartiles of AIP				Total (*n* = 1292)	*p* for Trend
	Q1 (*n* = 322) <−0.38	Q2 (*n* = 324) −0.38 to 0.09	Q3 (*n* = 323) 0.09 to 0.54	Q4 (*n* = 323) ≥0.54		
Grains (g)	329.2 ± 9.20	323.1 ± 10.5	333.7 ± 9.55	318.5 ± 9.48	326.1 ± 5.31	0.582
Potatoes and starches (g)	49.8 ± 9.04	47.0 ± 7.77	39.5 ± 6.43	46.0 ± 6.98	45.6 ± 4.74	0.582
Legumes and legume products (g)	41.6 ± 5.43	44.5 ± 6.39	48.4 ± 6.02	47.9 ± 7.51	45.6 ± 3.08	0.464
Nuts and seeds (g)	7.59 ± 2.93	9.14 ± 2.38	6.42 ± 1.80	5.61 ± 1.05	7.19 ± 1.18	0.349
Vegetables (g)	389.2 ± 15.8	383.3 ± 13.2	402.3 ± 14.6	376.8 ± 14.1	387.9 ± 7.93	0.758
Mushrooms (g)	5.90 ± 1.21	6.27 ± 1.17	6.23 ± 1.52	7.46 ± 1.53	6.47 ± 0.61	0.496
Fruits (g)	157.8 ± 14.0	192.3 ± 16.1	168.1 ± 16.5	159.4 ± 19.6	169.4 ± 9.03	0.791
Meats and poultries (g)	149.2 ± 11.2	174.2 ± 14.2	159.3 ± 11.9	150.0 ± 10.4	158.2 ± 6.20	0.788
Eggs (g)	37.6 ± 3.56	34.1 ± 3.10	35.9 ± 3.13	36.8 ± 4.25	36.1 ± 2.08	0.980
Fish and shellfish (g)	123.0 ± 11.8	104.7 ± 9.82	113.9 ± 8.90	113.9 ± 8.90	109.3 ± 11.3	0.524
Seaweed (g)	27.9 ± 5.56	20.6 ± 5.40	17.7 ± 3.12	25.2 ± 6.31	22.9 ± 3.12	0.684
Milk and dairy products (g)	131.9 ± 16.1	84.90 ± 9.74	97.1 ± 10.6	85.7 ± 12.3	99.8 ± 6.80	0.044
Oils and fats (g)	11.9 ± 0.83	10.8 ± 0.85	11.4 ± 0.92	12.0 ± 0.83	11.5 ± 0.45	0.816
Beverages and alcohols (g)	380.9 ± 35.6	535.7 ± 55.5	359.0 ± 36.0	460.4 ± 38.9	434.0 ± 24.5	0.680

**Table 5:** Contribution of seven major lipid-rich food groups toward the daily mean intake of lipid according to quartile of AIP (%) (adjusted energy intake, age, BMI, smoking, and physical activity).

	Quartiles of AIP				Total (*n* = 1292)	*p* for Trend
	Q1 (*n* = 322) <−0.38	Q2 (*n* = 324) −0.38 to 0.09	Q3 (*n* = 323) 0.09 to 0.54	Q4 (*n* = 323) ≥0.54		
Meats and poultries	25.5 ± 2.04	27.1 ± 2.21	23.9 ± 2.01	27.2 ± 2.44	25.9 ± 1.14	0.895
Grains	18.1 ± 1.70	18.2 ± 1.85	19.4 ± 1.80	14.2 ± 1.41	17.5 ± 0.93	0.165
Oils and fats	14.3 ± 1.41	13.7 ± 1.33	15.3 ± 1.25	17.3 ± 1.48	15.1 ± 0.72	0.116
Milk and dairy products	9.10 ± 1.48	6.17 ± 0.96	6.26 ± 1.04	4.26 ± 0.79	6.45 ± 0.65	0.007
Legumes and legume products	6.45 ± 0.97	6.60 ± 1.05	6.70 ± 0.91	5.03 ± 1.06	6.20 ± 0.55	0.338
Eggs	5.85 ± 0.73	5.54 ± 0.75	5.15 ± 0.64	7.71 ± 1.19	6.06 ± 0.49	0.223
Fish and shellfish	5.04 ± 0.61	4.56 ± 0.61	5.39 ± 0.61	4.88 ± 0.71	4.97 ± 0.33	0.889

## Data Availability

2013–2014 Sixth KNHANES data can be downloaded from the KNHANES homepage (https://knhanes.kdca.go.kr/knhanes/eng/index.do, accessed on 1 March 2022).

## Supplementary Materials

The following supporting information can be downloaded at https://www.mdpi.com/article/10.3390/nu14051071/s1: Table S1: Lipid intake of subfood in the group of milk and dairy products according to quartile of AIP (adjusted for energy intake, age, BMI, smoking, and physical activity).
