# Supplementary material for: Atherogenic Index of Plasma and Its Association with Risk Factors of Coronary Artery Disease and Nutrient Intake in Korean Adult Men: The 2013–2014 KNHANES
Source: Nutrients. 2022 Mar 3;14(5):1071. doi: 10.3390/nu14051071 (PMC8912761; doi:10.3390/nu14051071)
Supplement: Supplementary file 1 [file nutrients-14-01071-s001.zip › nutrients-1551998-supplementary.pdf]

Table S1. Lipid intake of subfood in the group of milk and dairy products according to quartile of AIP (adjusted for energy intake, age, BMI, smoking and physical activity).

	**Quartiles of AIP**						
	**Q1** **(*n* = 322)** **<−0.38**	**Q2** **(*n* = 324)** **−0.38 to 0.09**	**Q3** **(*n* = 323)** **0.09 to 0.54**	**Q4** **(*n* = 323)** **≥0.54**	**Total** **(*n* = 1292)**	***p*-Value**	***p* for Trend**
Milk (g)	2.85±0.48	1.73±0.22	1.82±2.97	1.49±0.38	2.04±0.18	0.035	0.009
Ice cream (g)	1.02±0.30	0.88±0.23	0.47±0.13	0.60±0.16	0.74±0.14	0.099	0.062
Yogurt (g)	0.31±0.08	0.16±0.06	0.17±0.05	0.11±0.05	0.19±0.03	0.291	0.075
Cheese (g)	0.03±0.02	0.18±0.05	0.14±0.04	0.17±0.07	0.13±0.03	0.074	0.117
Cream (g)	0.00±0.00	0.00±0.00	0.02±0.02	0.00±0.00	0.01±0.00	0.795	0.323
